# Optimal dietary arginine improves productive performance, gut morphology, and expression of growth-related and stress-response genes in Japanese quails

**DOI:** 10.1016/j.psj.2025.105684

**Published:** 2025-08-13

**Authors:** Ali Reza Ghiasvand, Hassan Shirzadi, Hossein Ali Ghasemi, Kamran Taherpour, Shokoufeh Hasanvand, Ali Khatibjoo

**Affiliations:** a Department of Animal Science, Faculty of Agriculture, Ilam University, Ilam, Iran; b Department of Animal Science, Faculty of Agriculture and Environment, Arak University, Arak, 38156-8-8349, Iran; c Department of Animal Science, Khorasan Razavi Agricultural and Natural Resources Research and Education Center, AREEO, Mashhad, Iran

**Keywords:** Arginine, Productive performance, Gut morphology, Gene expression, Japanese quails

## Abstract

This research examines the impact of different dietary arginine (Arg) concentrations on growth performance, carcass traits, gut morphology, and gene expression in Japanese quails aged 1 to 35 days. A total of 600 Japanese quail chicks were randomly allocated to five dietary treatments consisting of 0.75%, 1.0%, 1.25%, 1.50%, and 1.75% digestible Arg, with six replicate cages for each treatment and 20 birds housed per cage. Dietary digestible Arg concentrations of 1.0% or above led to improvements in growth performance, with the groups receiving 1.25% and 1.50% demonstrating the most pronounced increases in body weight and average daily gain (ADG) by day 35. The group receiving 1.25% digestible Arg had the most advantageous feed conversion ratio (FCR) and showed an increase in average daily feed intake. According to the one-slope linear broken-line regression model, the estimated breakpoint for ADG was 1.19% digestible Arg, while the breakpoint for FCR was 1.30% digestible Arg. Quadratic regression analysis further estimated maximal ADG at 1.42% digestible Arg and minimal FCR at 1.47% digestible Arg during the 1–35 day period. Furthermore, the 1.25% digestible Arg diet markedly improved gut morphology, especially in terms of jejunal villus height and surface area, which suggests an enhanced capacity for nutrient absorption. The group receiving 1.25% digestible Arg demonstrated superior carcass yield, significantly exceeding that of the 0.75% and 1.75% groups. Gene expression analysis revealed that hepatic HSP90 expression peaked in the 1.00% Arg group, but the expression levels of hepatic target of rapamycin (TOR) and ribosomal protein S6 kinase alpha-1 (RPS6KA1) were considerably elevated in the 1.50% and 1.75% groups. The treatments with 1.25% and 1.50% digestible Arg demonstrated the highest levels of kidney HSP70 expression. In conclusion, dietary supplementation at a concentration of 1.25% and more digestible Arg improved growth performance, carcass yield, gut morphology, and expression of genes related to growth and stress in Japanese quail, with model-based estimates refining the digestible Arg requirement range between 1.19% and 1.47%.

## Introduction

Arginine (Arg) is a vital amino acid that plays a crucial role in the physiological processes of chickens. This amino acid participates in multiple biological processes, such as protein synthesis, immunological function, and metabolic regulation (Khajali and Wideman, 2010; Hassan et al., 2021). Arginine functions as a precursor for nitric oxide, a molecule that improves blood circulation, nutrient transport, and muscle growth. Consequently, these characteristics have made Arg a significant subject of investigation within the realm of poultry nutrition (Liu et al., 2019; Ruan et al., 2020). Numerous studies have unequivocally demonstrated that supplementing poultry with Arg can improve growth, carcass quality, and overall health (Xu et al., 2018; Oliveira et al., 2022; Liu et al., 2023). However, in species such as Japanese quail, which have different metabolic and developmental characteristics from other common poultry, such as broilers and layers, further modifications in nutritional strategies are needed.

In addition to the proven effects of Arg on growth and carcass characteristics, recent research has shown that this amino acid has significant effects on the expression of genes involved in protein metabolism, especially genes related to muscle protein turnover. Two key targets in this process are the target of rapamycin (TOR) and ribosomal protein S6 kinase alpha-1 (RPS6KA1), both of which are essential for the regulation of protein synthesis and muscle growth (Flees et al., 2021; Ndunguru et al., 2024). TOR, which is at the core of the mTOR signaling pathway, regulates cell growth, protein synthesis, and metabolism in response to the availability of nutrients, including Arg (Yang et al., 2019). RPS6KA1, a downstream target of mTOR, plays a critical role in the translation of proteins required for muscle growth and overall development (Reda et al., 2024). Additionally, maintaining protein balance and supporting cellular stress responses rely on heat shock proteins (HSPs), especially HSP70 and HSP90. In stressful conditions, HSP70 acts as a molecular chaperone, facilitating the proper folding of new proteins and aiding in the repair of damaged ones. HSP90 plays a crucial role in the correct folding and optimal functioning of proteins, aiding in their stability throughout cellular processes and during protein synthesis (Lei et al., 2009). Elevated levels of HSP70 and HSP90 are associated with the presence of amino acids, especially Arg, and enhance metabolic activities as well as cellular resistance to stress (Xia et al., 2019; Mao et al., 2024). Furthermore, the kidneys are the primary site for the expression of the arginase 2 (ARG2) gene, which is essential for the conversion of Arg to ornithine, thereby having a significant impact on detoxification and nitrogen metabolism. Studies indicate that Arg supplementation can modulate ARG2 expression in both the liver and kidneys, contributing to the maintenance of metabolic balance (Uyanga et al., 2023).

While considerable research has explored the metabolic functions of Arg, the precise influence it exerts on the expression of genes associated with protein metabolism and stress responses in quail is still not well understood. This study aimed to explore the impact of varying digestible Arg levels (ranging from 0.75% to 1.75%) in the diet on growth performance, carcass characteristics, intestinal morphology, and the expression of genes associated with growth and stress in Japanese quail. This study seeks to bridge existing scientific gaps by determining the optimal Arg levels in the diet for enhancing production performance and physiological traits in meat-type quail.

## Materials and methods

### Ethical statement

This study was conducted in accordance with established ethical standards and was approved by the Animal Ethics Committee of Ilam University (Approval No.: 9815110702). All procedures followed the guidelines outlined in the Guide for the Care and Use of Experimental Animals.

#### Birds and Diets

A total of 600 unsexed, one-day-old Japanese quail chicks (Coturnix coturnix japonica) were procured from a local hatchery, with the parent stock being 160 days old. Upon arrival, the weight of each chick was measured individually and subsequently randomly assigned to one of 30 cages designated for five dietary treatments, each with six replicates. Cages were organized to reduce initial body weight variation, ensuring that the maximum variation in body weight among the groups did not exceed 0.2 g. The duration of the experimental period was 35 days, commencing on day 1 following hatching. The quail were maintained in wire-floored cages measuring 60 × 55 × 35 cm, which were fitted with electric bulbs to provide supplemental heating. For the initial five days, continuous lighting was implemented, followed by the establishment of a regimen consisting of 23 hours of light per day. The ambient temperature was set at 35°C and subsequently reduced by 4°C each week, reaching a temperature of 27°C at the onset of the third week. The relative humidity was consistently controlled within the range of 50% to 60% during the duration of the trial.

A basal mash diet developed to fulfill all essential nutrient requirements for Japanese quail, with the exception of Arg. Crystalline L-arginine (Evonik Degussa, Germany) was integrated into the basal diet through the substitution of corn starch, resulting in dietary digestible Arg concentrations of 0.75%, 1.00%, 1.25%, 1.50%, and 1.75%, as outlined in Table 1. All dietary plans were formulated to ensure the equilibrium of other vital nutrients, as indicated in Table 2. Feed and water were provided ad libitum throughout the entire experimental period. The amino acid concentrations in the basal diet were calculated using standardized ileal digestible (SID) amino acid data from [Aminodat® 5.0 (Evonik Industries, Germany)].

**Table 1:** Feed Ingredients of basal diet (as fed basis).

Ingredients (% of the diet)	% of diet
Wheat, Red W.	55.00
Corn	15.98
Corn Gluten Meal	17.86
Soybean Meal	3.20
Corn Starch^1^	1.21
Soybean Oil	0.50
Dicalcium Phosphate	1.18
Calcium Carbonate	1.48
KHCO_3_	0.64
NaHCO_3_	0.34
NaCl	0.10
Mineral–vitamin premix^2^	0.50
L-Lysine-HCL	1.05
DL-Methionine	0.14
L-Threonine	0.23
L-Tryptophan	0.05
L-Valine	0.20
L-Isoleucine	0.12
Xylanase^3^	0.02
Toxin binder	0.20

1 The incremental levels of L-arginine supplementation in the experimental treatments were 0.01%, 0.26%, 0.51%, 0.76%, and 1.01% of the diet, respectively. These amounts replaced corn starch to achieve digestible arginine concentrations of 0.75%, 1.00%, 1.25%, 1.50%, and 1.75% of the diet, respectively.

2 The vitamin and mineral premix provide the following quantities per kilogram of diet: Vitamin A (retinyl acetate), 6600 IU; cholecalciferol, 2800 IU; vitamin E, 20 IU (α-tocopheryl); vitamin K3 (menadione dimethpyrimidinol, 3.0 mg; riboflavin, 18.0 mg; niacin, 50 mg; pantothenic acid, 24 mg; biotin, 8.8 mg; choline chloride, 450 mg; vitamin B12, 0.02 mg; folic acid, 3.0 mg; manganese (MnO), 75 mg; zinc (ZnO), 100 mg; iron (FeSO_4_), 60 mg; copper (CuSO_4_), 12 mg; selenium (Na_2_SeO_3_), 0.2 mg; antioxidant, 250 mg.

3 Commercial xylanase (Nutrex NV., Achrerstenhoek. Belgium) with activity of 1500 units per g of product).

**Table 2:** Calculated and analyzed chemical composition of basal diet.

	Dietary arginine levels (%)				
Item	0.75	1.00	1.25	1.50	1.75
Calculated analysis (% of diet, unless otherwise stated)					
ME, Kcal/kg	2,950	2,950	2,950	2,950	2,950
Crude protein	23.2	23.2	23.2	23.2	23.2
Calcium	0.85	0.85	0.85	0.85	0.85
Available phosphorus	0.32	0.32	0.32	0.32	0.32
SID^1^ lysine	1.25	1.25	1.25	1.25	1.25
SID methionine + cysteine	0.88	0.88	0.88	0.88	0.88
SID arginine	0.75	1.00	1.25	1.50	1.75
SID threonine	0.78	0.78	0.78	0.78	0.78
SID tryptophan	0.19	0.19	0.19	0.19	0.19
SID valine	1.02	1.02	1.02	1.02	1.02
SID isoleucine	0.86	0.86	0.86	0.86	0.86
DEB^2^, mEq/kg	240	240	240	240	240
Analyzed values^3^ (% of diet)					
Total lysine	1.37 ± 0.05	1.38 ± 0.06	1.37 ± 0.04	1.38 ± 0.04	1.39 ± 0.03
Total methionine	0.63 ± 0.02	0.58 ± 0.03	0.60 ± 0.01	0.59 ± 0.03	0.58 ± 0.04
Total methionine + cysteine	1.02 ± 0.05	1.00 ± 0.04	1.01 ± 0.05	1.00 ± 0.06	1.02 ± 0.04
Total arginine	0.86 ± 0.03	1.12 ± 0.05	1.35 ± 0.04	1.61 ± 0.06	1.87 ± 0.06
Total threonine	0.90 ± 0.03	0.90 ± 0.02	0.89 ± 0.04	0.89 ± 0.03	0.90 ± 0.02
Total tryptophan	0.22 ± 0.005	0.26 ± 0.004	0.26 ± 0.003	0.27 ± 0.005	0.26 ± 0.004
Total tyrosine	0.88 ± 0.03	0.87 ± 0.02	0.89 ± 0.03	0.88 ± 0.04	0.87 ± 0.03
Total valine	1.20 ± 0.05	1.20 ± 0.04	1.21 ± 0.04	1.20 ± 0.02	1.22 ± 0.03
Total isoleucine	1.03 ± 0.05	1.05 ± 0.03	1.01 ± 0.03	1.05 ± 0.04	1.02 ± 0.04
Total leucine	2.61 ± 0.07	2.58 ± 0.06	2.58 ± 0.05	2.60 ± 0.07	2.61 ± 0.05
Total histidine	0.41 ± 0.01	0.41± 0.02	0.41± 0.01	0.41± 0.02	0.41± 0.01

1 Standardized ileal digestible

2 DEB (dietary electrolyte balance) = (Na^+^, mEq/kg + K^+^, mEq/kg) – CL^−^, mEq/kg.

3 Mean and standard deviation of three samples per diet.

### Chemical Analysis

The determination of the chemical composition of the protein sources and experimental diets was conducted in accordance with the procedures outlined by AOAC (2007). The parameters evaluated encompassed dry matter, crude fat, crude protein, crude fiber, crude ash, calcium, total phosphorus, and nitrogen-free extract. The determination of crude protein content was made through the quantification of nitrogen, employing a conversion factor of 6.25. The analysis of amino acid profiles was conducted utilizing an automatic amino acid analyzer (L-8800, Hitachi, Tokyo, Japan). Before analysis, the samples were hydrolyzed in 6M hydrochloric acid at a temperature of 110°C for a duration of 24 hours. Before hydrolysis, performic acid oxidation was performe/d for sulfur-containing amino acids.

### Growth Performance, Carcass Evaluation, and Sampling

Growth performance was evaluated over a period of 35 days during the trial. Body weight (BW) was measured on days 1 and 35, with mean values computed for each cage. The average daily feed intake (ADFI) was assessed on a per-cage basis, while the average daily gain (ADG) was calculated. The feed conversion ratio (FCR) was calculated as the ratio of average daily feed intake (ADFI) to average daily gain (ADG). Daily monitoring of mortality was conducted, and performance metrics were modified as necessary.

On day 35, after a three-hour period of feed withdrawal, two quails from each cage were randomly selected for the purposes of slaughter and subsequent carcass evaluation. Carcass traits were evaluated by measuring the weights of boneless, skinless breast and leg meat, as well as various internal organs, including the liver, heart, gizzard, small intestine, bursa, and spleen. The weights were recorded immediately after death and expressed as percentages of the live body weight.

After the experiment was completed, tissue samples were systematically collected. Birds were euthanized by cervical dislocation, followed by the harvesting of the jejunum for histological analysis. Liver and kidney tissues were harvested and promptly preserved at –80°C for future gene expression analysis.

### Histological Study of Jejunal Structure

From each bird, jejunal tissue samples approximately 2 cm in length were collected and preserved in 10% neutral-buffered formalin for 24 to 48 hours. Using a rotary microtome, samples were dehydrated, embedded in paraffin, and then sectioned into 5 μm slices. Using a light microscope (BX53, Olympus, Tokyo, Japan), sections were stained with hematoxylin and eosin (H&E) and then inspected. The evaluation focused on key morphological characteristics to assess intestinal growth and absorptive capacity, including villus height (VH), crypt depth (CD), villus width (VW), villus surface area (VSA), and the ratio of villus height to crypt depth (VH/CD). Images were acquired with a digital camera (DP72, Olympus, Tokyo, Japan), and subsequent measurements were performed using image analysis software (QWinPlus, version 3.1.0; Leica Cambridge Ltd., UK). For each bird, a minimum of 10 well-oriented villi from various jejunal sections were measured to ensure accuracy and representativeness.

### RNA Isolation and cDNA Synthesis

Using TRIzol reagent (Invitrogen, Carlsbad, CA, USA), total RNA was isolated from liver and kidney tissues following the manufacturer's instructions. A NanoDrop spectrophotometer (Thermo Fisher Scientific, Waltham, MA, USA) was used to assess the concentration and purity of RNA; agarose gel electrophoresis verified these findings. First-strand cDNA was synthesized from 1 μg total RNA using the High-Capacity cDNA Reverse Transcription Kit (Thermo Fisher Scientific) and stored at –20°C until required for further applications. Quantitative real-time PCR (qRT-PCR) was performed to assess the expression levels of target genes, specifically TOR, RPS6KA1, HSP70, and HSP90 in liver tissues, as well as ARG2, HSP70, and HSP90 in kidney tissues. The primer sequences were developed using Oligo (version 7.56) software. The primer sequences are detailed in Table 3. Gene expression levels were normalized using glyceraldehyde-3-phosphate dehydrogenase (GAPDH) as the internal reference gene. The cycling conditions for qRT-PCR were as follows: an initial denaturation step at 95°C for 3 minutes, followed by 40 cycles comprising denaturation at 95°C for 15 seconds and annealing/extension at 60°C for 60 seconds. The reaction mixture, with a total volume of 20 μL, comprised 1 × SYBR Green PCR Master Mix, double-distilled water, 0.2–0.4 μM of each primer, and 50–100 ng of cDNA template. Relative gene expression levels were determined using the 2^–ΔΔCt^ method.

**Table 3:** Gene Expression Primers.

Gene^1^	Forward primer	Reverse primer	Gene bank accession number	Product size (bp)
TOR	5′-GTGGCAAGAGAGCAGGAGTGAG-3′	5′-GTCAGTTGTGTAGGTCCAATCATAAGG-3′	XM_046909668	76
RPS6KA1	5′-CACTACGCATTCCAGACAGAG-3′	5′-GGTCAAGTCCAAGAGCAAGC-3′	XM_046931819	148
HSP70	5′-AGTGGCTGACTGACCAGAGGA-3′	5′-CAAGAATACGTGGTGCCCAGA-3′	NM_001006685	84
HSP90	5′-GCATTCTCAGTTCATTGGCTACC-3′	5′-TTCATCATCACTCACCTCCTTATCG-3′	NM_001109785	76
ARG2	5′-GCTGATATTAACACTCCTCTTACAACTC-3′	5′-ATCCACATCCCTCAACCCAATG-3′	NM_001199704	171
GAPDH	5′-CAGAACATCATCCCAGCGTCCAC-3′	5′-CGGCAGGTCAGGTCAACAACAG-3′	NM_204305	134

1 TOR, target of rapamycin; RPS6KA1, ribosomal protein S6 kinase polypeptide 1; HSP70, heat shock protein 70; HSP90, heat shock protein 90; ARG2, arginase 2; GAPDH, glyceraldehyde-3-phosphate dehydrogenase.

### Statistical Analysis

Data were analyzed using one-way analysis of variance (ANOVA), followed by Tukey’s post hoc test for multiple comparisons. Gene expression results were expressed as fold changes relative to the control group (0.75% Arg). Statistical significance was considered at p < 0.05. All analyses were performed using SAS software (version 9.2; SAS Institute Inc., Cary, NC, USA). The dietary digestible Arg requirements were estimated utilizing broken-line methodology (Robbins et al., 2006) and by fitting the data to a quadratic response curve (Draper and Smith, 1981). The linear broken-line model was defined as response = L + U × (R − x), where L denotes the plateau, U is the rate constant, R represents the requirement, and x is the dietary digestible Arg level. The equation for the quadratic regression model was expressed as response = a + bx + cx², where a, b, and c are regression parameters, and x represents the dietary Arg level. A value corresponding to 95% of the X-value required for maximal Y-value for ADG and minimum Y-value for FCR was selected as a subjective estimate of the requirement.

## RESULTS

### Growth Performance

Table 4 presents the growth performance data for Japanese quails from day 1 to day 35. The results indicate that the groups receiving 1% or higher digestible Arg had significantly greater BW at day 35 and ADG from day 1 to day 35 compared to the 0.75% digestible Arg group (P < 0.05). Specifically, the highest BW and ADG were observed in the 1.25% and higher digestible Arg groups (P < 0.05). Throughout the experimental period, the 1.25% and 1.50% digestible Arg treatments resulted in significantly higher ADFI compared to the 0.75% digestible Arg group (P < 0.05). Additionally, the FCR from days 1 to 35 was significantly lower (P < 0.05) in the groups receiving 1% digestible Arg or more, with the lowest total FCR found in the 1.25% and higher digestible Arg treatments.

**Table 4:** Performance parameters^1^ of Japanese quails fed different dietary arginine levels from 1 to 35 days of age.

	Dietary arginine levels (%)						*P*-value		
	0.75	1.00	1.25	1.50	1.75	SEM	Treatments	Linear	Quadratic
Body weight, g	172.1^c^	181.4^b^	188.2^a^	191.1^a^	187.3^a^	0.97	<0.001	<0.001	<0.001
ADG, g/bird/d	4.71^c^	4.97^b^	5.16^a^	5.25^a^	5.14^a^	0.028	<0.001	<0.001	<0.001
ADFI, g/bird/d	14.62^b^	14.89^ab^	14.99^a^	14.97^a^	14.90^ab^	0.082	0.028	0.020	0.016
FCR	3.11^a^	2.99^b^	2.90^c^	2.85^c^	2.90^c^	0.015	<0.001	<0.001	<0.001

Means within a row showing different superscripts are significantly different (*P*< 0.05). Values are means of 6 cages per treatment combination with 20 quail chicks

1 ADG, average daily gain; ADFI, average daily feed intake; FCR, feed conversion ratio

**Figure 1:**
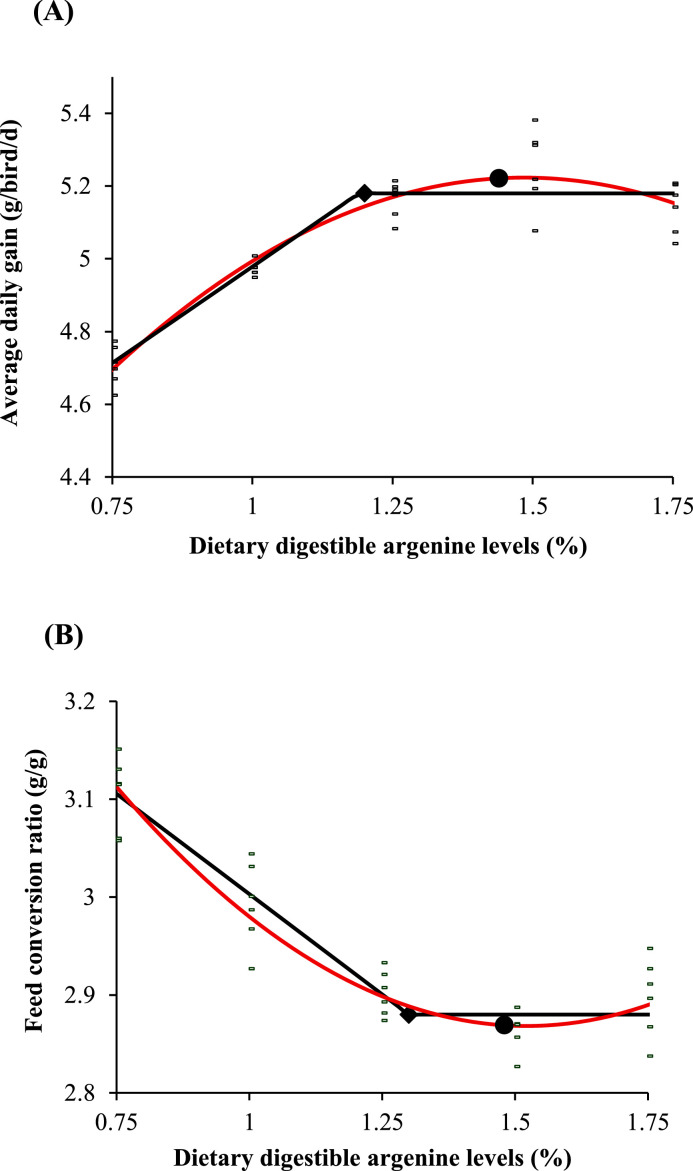
Fitted broken line and quadratic regression models of average daily gain (A; ADG) and feed conversion ratio (B; FCR) for 1–35-day period as a function of dietary arginine content. The minimal digestible arginine requirement determined by the one-slope linear broken line analysis (black line) was 1.19% [Y = 5.18 – 1.06 (1.19 – X); *r*^2^ = 87.4%] for ADG and 1.30% [Y = 2.88 + 0.41 (1.30 – X); *r*^2^ = 85.2%] for FCR. The cage means data from Table 4 also were fitted to a quadratic regression equation (Red line): [Y = 3.818 - 1.248 X + 0.410 X^2^; *r*^2^ = 89.9 %] for ADG and [Y = 3.067 + 2.906 X – 0.979 X^2^; *r*^2^ = 86.6 %] for FCR. The level of dietary digestible arginine that maximized ADG (i.e., upper asymptote) was calculated to be 1.42% and the level that minimized FCR was 1.47%.

Figure 1 illustrates the fitted one-slope linear broken-line and quadratic regression models for ADG and FCR during the 1-35-day period as a function of digestible dietary Arg. The single-slope linear broken-line analysis identified breakpoints at 1.19% for ADG (Figure 1a) and 1.30% digestible Arg for FCR (Figure 1b). The quadratic regression equations indicated that the digestible Arg levels required to achieve maximal ADG (upper asymptote) was 1.42% and the digestible Arg levels required to achieve minimum FCR (lower asymptote) was 1.47% during the 1-35-day period.

#### Slaughter-Related Traits

Table 5 summarizes the slaughter-related traits, including carcass characteristics and the relative weights of visceral organs in quails at 35 days of age. The experimental treatments had no significant effect on breast and leg meat yields or the relative weights of organs, including the liver, heart, gizzard, small intestine, bursa of Fabricius, and spleen (P > 0.05). However, the treatments significantly influenced carcass yield in both linear and quadratic trends (P < 0.05). While the 1.50% digestible Arg treatment resulted in a higher carcass yield than the 0.75% treatment, the 1.25% digestible Arg treatment produced the greatest carcass yield, which was significantly higher than the 0.75% and 1.75% treatments (P < 0.05).

**Table 5:** Slaughter traits^1^ of Japanese quails fed different dietary arginine levels .

	Dietary arginine levels (%)						*P*-value		
Item	0.75	1.00	1.25	1.50	1.75	SEM	Treatments	Linear	Quadratic
Day 35									
Carcass	61.43^c^	62.59^abc^	63.91^a^	63.24^ab^	62.31^bc^	0.341	0.001	0.036	<0.001
Breast	26.24	26.47	26.45	26.87	25.19	0.928	0.761	0.568	0.340
Leg	15.14	14.55	15.36	15.24	14.45	0.391	0.359	0.592	0.369
Heart	0.870	0.790	0.838	0.837	0.882	0.051	0.752	0.670	0.311
Liver	2.46	2.61	2.93	2.69	2.99	0.197	0.316	0.081	0.724
Gizzard	1.78	1.76	1.80	1.77	1.74	0.093	0.994	0.819	0.793
Small intestine	4.33	4.53	4.19	4.13	4.49	0.535	0.978	0.957	0.766
Bursa	0.099	0.082	0.124	0.099	0.099	0.011	0.142	0.624	0.421
Spleen	0.084	0.083	0.066	0.073	0.091	0.013	0.641	0.931	0.190

Means within a row showing different superscripts are significantly different (*P*< 0.05). Values are means of 6 cages per treatment combination with 2 male quail chicks per cage.

1 Based on preslaughter body weight (%)

#### Gut morphology

Table 6 shows the jejunal morphological parameters. Dietary treatments had no significant effect on CD or the VH/CD ratio (P > 0.05). In contrast, quails fed the 1.25% digestible Arg diet had significantly greater VH (quadratic, P < 0.05) compared to those on the 0.75% digestible Arg diet. Similarly, quails on the 1.25% digestible Arg diet exhibited significantly greater VSA (quadratic, P < 0.05) compared to those on the 0.75% and 1.75% digestible Arg diets. Birds on the 1.25% and 1.50% digestible Arg diets also showed a tendency towards greater VW (P = 0.077).

**Table 6:** Jejunal morphology of Japanese quails fed different dietary arginine levels.

	Dietary arginine levels (%)						*P*-value		
Item	0.75	1.00	1.25	1.50	1.75	SEM	Treatments	Linear	Quadratic
Villus height, µm	579.3^b^	646.9^ab^	779.5^a^	701.7^ab^	635.7^ab^	42.02	0.028	0.219	0.006
Villus width, µm	97.3	99.0	107.0	105.3	89.7	4.46	0.077	0.527	0.014
Crypt depth, µm	71.94	67.30	82.53	80.91	68.63	5.26	0.163	0.679	0.116
VH/CD^1^	8.08	9.65	9.54	8.90	9.54	0.644	0.397	0.294	0.334
VSA^2^, mm^2^	0.178^b^	0.203^ab^	0.264^a^	0.235^ab^	0.178^b^	0.0198	0.019	0.636	0.002

Means within a row showing different superscripts are significantly different (*P*< 0.05). Values are means of 6 cages per treatment combination with 2 male quail chicks per cage.

1 VH/CD, villus height to crypt depth ratio

2 VSA, Villus surface area = 2π × (VW/2) × VH

**Figure 2:**
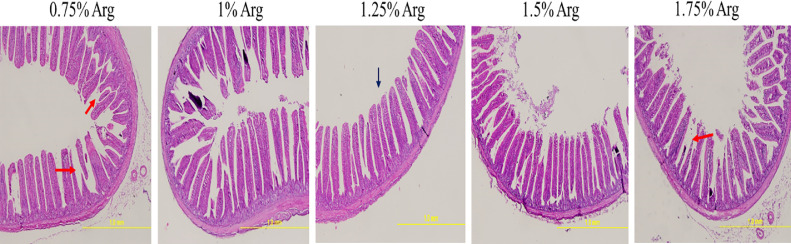
The histological features of the mucosa of jejunum based on hematoxylin and eosin staining in 35-days-old Japanese quails fed with different dietary digestible arginine (Arg) levels, images at a lower magnification (100 ×) are provided. Red arrows indicate incomplete villi structures, while Black arrows denote complete and developed ones.

Histological analysis of the jejunal mucosa (Figure 2) confirmed these findings. The mucosal villi in the 1.25% and 1.50% digestible Arg groups were consistently longer and more closely packed compared to the 0.75% digestible Arg group. The intestinal morphology was notably improved in the 1.25% and 1.50% digestible Arg groups, reflecting enhanced structural integrity and absorptive capacity.

#### Gene expression

**Figure 3:**
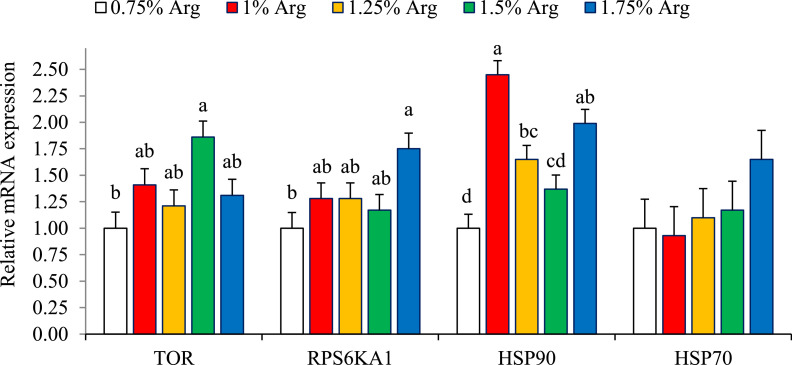
Bar charts of hepatic mRNA expression levels of target of rapamycin (TOR), ribosomal protein S6 kinase polypeptide 1 (RPS6KA1), heat shock protein 90 (HSP90), and heat shock protein 70 (HSP70) in 35-day-old Japanese quails fed different dietary digestible arginine (Arg) levels. ^a–d^ Different letters in the same histogram indicate significant differences among groups according to Tukey's multiple range test (P < 0.05). Each bar represents the mean values and standard errors representing 6 replicates (cages) per treatment.

**Figure 4:**
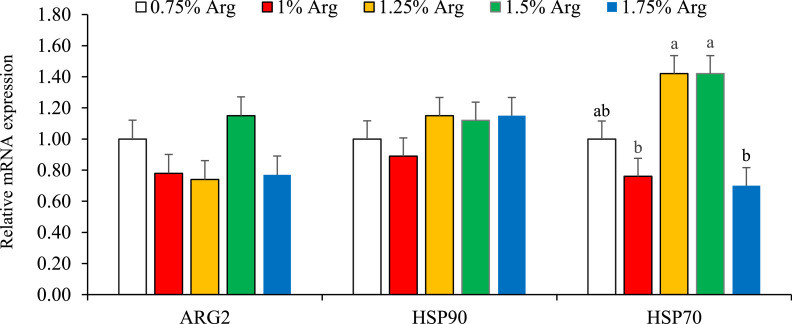
Bar charts of mRNA expression levels of renal arginase 2 (ARG2), heat shock protein 90 (HSP90), and heat shock protein 70 (HSP70) in 35-day-old Japanese quails fed different dietary digestible arginine (Arg) levels. Different letters in the same histogram indicate significant differences among groups according to Tukey's multiple range test (P < 0.05). Each bar represents the mean values and standard errors representing 6 replicates (cages) per treatment.

Figures 3 and 4 present the gene expression data for hepatic and kidney tissues at 35 days of age. Increasing dietary Arg levels had a linear effect on hepatic TOR expression, with the 1.50% digestible Arg treatment showing the highest TOR expression levels compared to the 0.75% digestible Arg treatment (P < 0.05). The 1.75% Arg treatment also showed significantly higher RPS6KA1 expression compared to the 0.75% digestible Arg treatment (P < 0.05; linear). In terms of hepatic HSP90 expression, diets containing 1%, 1.25%, and 1.75% digestible Arg all resulted in higher HSP90 expression compared to the 0.75% digestible Arg treatment (P < 0.05), with the 1% digestible Arg group exhibiting the highest expression levels. However, no significant effect was observed on hepatic HSP70 expression (P > 0.05).

In kidney tissues (Figure 4), the treatments had no significant impact on ARG2 or HSP90 expression levels (P > 0.05). However, HSP70 expression levels were significantly influenced in a quadratic manner (P < 0.05), with the highest expression observed in the 1.25% and 1.50% digestible Arg groups, which were notably higher than those in the 1% and 1.75% digestible Arg treatments.

## Discussion

This study aimed to investigate the impact of incorporating digestible Arg into the diet of Japanese broiler quail and to evaluate its effects on their growth and physiological responses. The findings indicated that the groups given at least 1% digestible Arg experienced a notable rise in body weight and average daily gain compared to the group that received 0.75% digestible Arg. The groups that received 1.25% digestible Arg or more showed the most substantial growth and weight gain. The findings underscore the significant and direct influence of elevated Arg levels on accelerating growth processes in poultry. These results align with earlier studies that have consistently demonstrated the beneficial impact of Arg on body weight gain and average daily gain in poultry (Castro et al., 2019; Filho et al., 2021). Arginine seems to play a key role in essential metabolic processes, such as protein synthesis, muscle growth, and overall metabolic efficiency, all of which contribute directly to improved growth and weight gain (Fernandes et al., 2009; Xu et al., 2018). Throughout the experiment, the groups receiving 1.25% and 1.5% digestible Arg had notably higher ADFI than the group receiving 0.75% digestible Arg. This significant difference suggests that Arg may increase the metabolic demands of birds during rapid growth periods and could also help stimulate appetite. The findings align with those of Lisnahan et al. (2022), who demonstrated that Arg can enhance feed intake during these growth phases. Furthermore, a study conducted by Zampiga et al. (2018) revealed that elevated levels of Arg may enhance feed intake in broiler chickens, likely as a result of improved nutrient absorption and the heightened energy requirements associated with growth.

In terms of FCR, the groups receiving 1% or more digestible Arg demonstrated superior results compared to the 0.75% digestible Arg group. The enhancement in FCR was particularly notable in the groups that received digestible Arg at a concentration of 1.25% or greater. FCR serves as an essential indicator of production efficiency in poultry, contributing to lower feed costs and promoting more sustainable farming practices. The beneficial impact of Arg on feed conversion ratio is likely due to its contribution to muscle growth, enhancement of nutrient metabolism, and improvement in protein synthesis, essential elements that ultimately result in increased feed efficiency (Ebrahimi et al., 2014; Castro et al., 2019).

Regarding model-based estimation of digestible Arg requirements, the present study employed regression analyses to refine dietary Arg levels that optimize growth performance and feed efficiency. The one-slope linear broken-line regression model estimated the breakpoint for ADG at approximately 1.19% digestible Arg and for FCR at 1.30% digestible Arg. Quadratic regression analysis further identified maximal ADG and minimal FCR at 1.42% and 1.47% digestible Arg, respectively, over the 1–35-day growth period. These refined estimates align with findings from Ali et al. (2025), who demonstrated that in broilers, precise Arg supplementation enhances growth and beneficially modulates nutrient metabolism, whereas excessive Arg reduces feed intake and growth due to metabolic imbalance and oxidative stress. Therefore, our results emphasize the importance of accurate Arg dosing to maximize quail performance and health, underscoring the dose-dependent nature of Arg responses.

These results suggest that multiple factors contributed to the improvements in feed efficiency and growth seen in poultry after Arg supplementation. Arginine is crucial for many important metabolic processes, including protein turnover, energy metabolism, and immune function. It also plays a vital role in reducing stress in birds, which is particularly valuable in the demanding environment of poultry production (De Souza Castro and Kim, 2020; Fathima et al., 2024b). A study conducted by Oliveira et al. (2022) confirmed that the inclusion of Arg in poultry diets significantly enhances metabolic efficiency and gut health, resulting in improved growth and performance. These findings highlight the promise of Arg as a valuable nutritional supplement for improving feed efficiency and growth in poultry. Such supplementation could play a significant role in advancing more sustainable and efficient poultry farming practices.

The results of this study showed a significant improvement in carcass yield. The 1.25% digestible Arg treatment achieved the highest yield, which was significantly higher than the 0.75% and 1.75% digestible Arg treatments. These findings clearly indicate that the use of Arg supplementation at medium to high levels is particularly effective in increasing carcass muscle mass, thereby improving production efficiency. These results are consistent with previous studies showing that Arg supplementation can improve carcass characteristics by increasing protein synthesis and supporting muscle growth (Miao et al., 2017; Pirsaraei et al., 2018). In other words, Arg influences particular muscle groups while also promoting overall muscle growth and enhancing body composition. The noted enhancement in carcass yield is linked to the significant function of Arg in protein metabolism and muscle development processes (Fathima et al., 2024a; Hassan et al., 2022). Arginine, serving as a precursor to nitric oxide, effectively enhances muscle growth by boosting blood flow and facilitating nutrient delivery to muscle tissues (Miri et al., 2022). Furthermore, Arg plays a crucial role in activating the mTOR signaling pathway, which is essential for regulating protein synthesis and facilitating muscle hypertrophy (Pirsaraei et al., 2018; Wang et al., 2022). These metabolic pathways probably contributed significantly to the enhanced muscle deposition and carcass yield observed in the 1.25% digestible Arg treatment. Additionally, the observation of a quadratic response in carcass yield indicates that the effects of Arg are dose-dependent, with a dose of 1.25% recognized as the optimal level to enhance muscle deposition. These findings are consistent with previous studies that have reported reduced carcass yield at very high levels of Arg supplementation (Zampiga et al., 2018; Westreicher-Kristen et al., 2025), emphasizing the importance of determining the correct balance of Arg supplementation to optimize muscle growth.

This study's results indicated that incorporating Arg into the quail diet, particularly at a dosage of 1.25%, notably enhanced jejunal morphology. This enhancement was evidenced by increases in villus height, width, and surface area, which contributed to improved nutrient absorption and overall gut health. These findings are consistent with previous studies that showed that Arg supplementation improved gut function and villi structure in broiler chickens (Yu et al., 2018; Brugaletta et al., 2023). Interestingly, Kheiri and Landy (2019) concluded that although higher Arg intake improved specific gut characteristics in Japanese quail, its effect on the overall gut structure was very limited under ideal conditions. This indicates that the beneficial impacts of Arg might be more significant during stressful situations or in species with greater metabolic requirements (Khajali et al., 2014; Castro et al., 2020; Kalvandi et al., 2022). The influence of Arg is likely affected by various factors, including the specific nutritional needs of each species, environmental conditions, and the composition of the basal diet. The quadratic response observed in VH and VSA at 1.25% digestible Arg supports the hypothesis that moderate Arg intake is most appropriate for intestinal development, as higher levels (1.75%) did not result in further improvement, suggesting that there is a threshold after which increasing Arg intake may be ineffective. This trend is consistent with previous studies in broilers showing that moderate Arg intake has the greatest positive effects (Fathima et al., 2024b; Brugaletta et al., 2023). Furthermore, these results are consistent with research showing that high Arg intake does not improve intestinal morphology (Lisnahan et al., 2022). Histologically, these results were confirmed by showing longer and denser villi in the 1.25% digestible Arg group, indicating improved intestinal structure. Intestinal health and the ability to absorb nutrients depend on such improvements in intestinal structure. Other research has also highlighted the role of Arg in strengthening the intestinal mucosa through cell growth and differentiation (Kheiri & Landy, 2019; Murakami et al., 2012).

The results indicated a linear increase in the expression of the TOR and RPS6KA1 genes in the liver as the dietary level of Arg increased. The group with 1.75% digestible Arg exhibited the highest expression of the RPS6KA1 gene compared to the group with 0.75% digestible Arg, whereas the highest expression of the TOR gene was observed in the 1.5% digestible Arg group. These findings indicate that Arg acts as a major stimulator in the process of protein synthesis through the TOR pathway. The TOR pathway plays a fundamental role in the regulation of protein metabolism and cell growth. These results are consistent with previous studies that have shown that Arg increases protein turnover through mTOR signaling. This effect depends on the amount of Arg in the diet and the tissue type (Yuan et al., 2015; Miao et al., 2017).

At 1% digestible Arg, the expression of HSP90, an accessory protein involved in stress responses, was highest. Expression also increased at 1.25% and 1.75% digestible Arg. However, the responses of the kidneys differed. ARG2 expression remained constant across all treatments, while HSP70 showed a quadratic response, peaking at 1.25% and 1.5% digestible Arg doses. The effect of Arg on HSP70 expression in the kidneys appears nonlinear and dose-dependent, with ideal doses between 1.25% and 1.5%. Previous studies have indicated that dietary Arg significantly affects heat shock proteins in various tissues under stress conditions (Hu et al., 2016; Xia et al., 2019). Previous studies (Hassan et al., 2021; Hu et al., 2016) have demonstrated that excessive levels of Arg impair immune function. In addition to its health benefits, research has shown that taking Arg can boost immune function and improve the body's ability to fight off damage from free radicals in broiler chickens. This leads to enhanced innate immune responses and increased antioxidant enzyme activity (Wang et al., 2022). Furthermore, Arg serves as a precursor for nitric oxide production, playing a crucial role in vasodilation and enhancing stress responses. This, in turn, contributes to better thermoregulation and improved immune function in birds (Uyanga et al., 2021). Interestingly, while Arg supplementation led to a significant reduction in HSP70 expression in the kidneys, only a minor increase was noted in the liver, and there was no consistent response to elevated digestible Arg levels. The results highlight that the influence of Arg on stress responses is contingent upon the composition of the diet and the characteristics of the tissue.

## Conclusions

In conclusion, diets containing 1% or more digestible Arg were associated with significant improvements in growth performance and carcass yield in Japanese quails. With the lowest feed conversion ratio, quails consuming 1.25% digestible Arg showed greater body weight, average daily gain, and feed intake, suggesting better feed efficiency. The 1.25% digestible Arg diet demonstrated superior efficacy in enhancing carcass yield compared to other tested levels, indicating its potential as a beneficial strategy to support production. Additionally, 1.25% digestible Arg supplementation also improved gut health as shown by increased jejunal villus height and surface area, therefore promoting better nutrient absorption. However, increased dietary digestible Arg at levels of 1.25% to 1.75% enhanced the expression of key proteins related to stress response and protein synthesis, including TOR, RPS6KA1, and HSP70. Based on the regression models used in this study, supplementing within the range of 1.2% to 1.5% digestible Arg offers a statistically supported and effective strategy for poultry producers to potentially enhance growth rates, improve feed utilization, and increase overall production efficiency.

## CRediT authorship contribution statement

**Ali Reza Ghiasvand:** Writing – original draft, Software, Investigation, Conceptualization. **Hassan Shirzadi:** Writing – review & editing, Writing – original draft, Supervision, Methodology, Funding acquisition, Formal analysis. **Hossein Ali Ghasemi:** Writing – original draft, Validation, Supervision, Software, Formal analysis, Data curation. **Kamran Taherpour:** Writing – review & editing, Writing – original draft, Visualization, Project administration, Investigation. **Shokoufeh Hasanvand:** Writing – review & editing, Writing – original draft, Software, Investigation, Data curation. **Ali Khatibjoo:** Writing – review & editing, Writing – original draft, Resources, Methodology, Conceptualization.

## Disclosures

The authors have no financial conflicts of interest.

## References

[bib0001] Ali A., Liu X., Melaku M., lqbal W., Bao Y., Ruqing Z., Chen L., Ma T., Zhang H. (2025). Effect of arginine supplementation on liver and pectoral muscle: tissue-specific lipid metabolism in broilers. Poult. Sci. In Press..

[bib0002] Hortwitz W., Latimer G.W., AOAC International (2007). Official Methods of Analysis.

[bib0003] Brugaletta G., Zampiga M., Laghi L., Indio V., Oliveri C., De Cesare A., Sirri F. (2023). Feeding broiler chickens with arginine above recommended levels: effects on growth performance, metabolism, and intestinal microbiota. J. Anim. Sci. Biotechnol..

[bib0004] Castro F.L.S., Su S., Choi H., Koo E., Kim W.K. (2019). L-arginine supplementation enhances growth performance, lean muscle, and bone density but not fat in broiler chickens. Poult. Sci..

[bib0005] Castro F.L.S., Teng P.-Y., Yadav S., Gould R.L., Craig S., Pazdro R., Kim W.K. (2020). The effects of L-Arginine supplementation on growth performance and intestinal health of broiler chickens challenged with *Eimeria* spp. Poult. Sci..

[bib0006] De Souza Castro F.L., Kim W.K. (2020). Secondary functions of arginine and sulfur amino acids in poultry health: review. Animals.

[bib0007] Draper N.R., Smith H. (1981).

[bib0008] Ebrahimi M., Shahneh A.Z., Shivazad M., Pirsaraei Z.A., Tebianian M., Ruiz-Feria C.A., Adibmoradi M., Nourijelyani K., Mohamadnejad F. (2014). The effect of feeding excess arginine on lipogenic gene expression and growth performance in broilers. Br. Poult. Sci..

[bib0009] Fathima S., Al Hakeem W.G., Shanmugasundaram R., Selvaraj R.K. (2024). Effect of arginine supplementation on the growth performance, intestinal health, and immune responses of broilers during necrotic enteritis challenge. Poult. Sci..

[bib0010] Fathima S., Hakeem W.G.A., Selvaraj R.K., Shanmugasundaram R. (2024). Beyond protein synthesis: the emerging role of arginine in poultry nutrition and host-microbe interactions. Front. Physiol..

[bib0011] Fernandes J.I.M., Murakami A.E., Martins E.N., Sakamoto M.I., Garcia E.R.M (2009). Effect of arginine on the development of the pectoralis muscle and the diameter and the protein:deoxyribonucleic acid rate of its skeletal myofibers in broilers. Poult. Sci..

[bib0012] Filho S.T.S., Da C Lima E.M., De Oliveira D.H., De Abreu M.L.T., Rosa P.V., De Laurentiz A.C., De P Naves L., Rodrigues P.B. (2021). Supplemental L-arginine improves feed conversion and modulates lipid metabolism in male and female broilers from 29 to 42 days of age. Animal.

[bib0013] Flees J.J., Emami N.K., Greene E., Ganguly B., Dridi S. (2021). Phytogenic water additives improve broiler growth performance via modulation of intermediary metabolism-related signaling pathways. Animals.

[bib0014] Hassan F., Arshad M.A., Hassan S., Bilal R.M., Saeed M., Rehman M.S. (2021). Physiological role of arginine in growth performance, gut health, and immune response in broilers: A review. World’s Poult. Sci. J..

[bib0015] Hassan S.S., Nassar A.N., Abd-Allah A.A., Abd EL-Hamid E.E. (2022). Effect of arginine supplementation on productive performance, carcass traits, hematology and economic efficiency of broilers under heat stress conditions. J. Agric. Environ. Sci..

[bib0016] Hu Y.-D., Tan J.-Z., Qi J., Zhang H.-F. (2016). Regulatory effects of dietary L-arg supplementation on the innate immunity and antioxidant ability in broiler chickens. J. Integr. Agric..

[bib0017] Kalvandi O., Sadeghi A., Karimi A. (2022). Arginine supplementation improves reproductive performance, antioxidant status, immunity and maternal antibody transmission in breeder Japanese quail under heat stress conditions. Ital. J. Anim. Sci..

[bib0018] Khajali F., Wideman R.F. (2010). Dietary arginine: metabolic, environmental, immunological and physiological interrelationships. World’s Poult. Sci. J..

[bib0019] Khajali F., Heydary Moghaddam M., Hassanpour H. (2014). An L-arginine supplement improves broiler hypertensive response and gut function in broiler chickens reared at high altitude. Int. J. Biometeorol..

[bib0020] Kheiri F., Landy N. (2019). Growth performance, intestinal morphology, serum biochemical and hematological parameters in Japanese quail (*Coturnix japonica*) fed supplemental L-Arginine. Braz. J. Poult. Sci..

[bib0022] Lei L., Yu J., Bao E. (2009). Expression of heat shock protein 90 (Hsp90) and transcription of its corresponding mRNA in broilers exposed to high temperature. Br. Poult. Sci..

[bib0023] Lisnahan C.V., Nahak O.R., Welsiliana W., Pardosi L. (2022). Effect of L-arginine and L-lysine HCl ratio on growth performance and ileum morphology of native chickens aged 2–14 weeks. Vet. World.

[bib0024] Liu G., Ajao A.M., Shanmugasundaram R., Taylor J., Ball E., Applegate T.J., Selvaraj R., Kyriazakis I., Olukosi O.A., Kim W.K. (2023). The effects of arginine and branched-chain amino acid supplementation to reduced-protein diet on intestinal health, cecal short-chain fatty acid profiles, and immune response in broiler chickens challenged with Eimeria spp. Poult. Sci..

[bib0025] Liu S., Tan J., Hu Y., Jia X., Kogut M.H., Yuan J., Zhang H. (2019). Dietary l-arginine supplementation influences growth performance and B-cell secretion of immunoglobulin in broiler chickens. J. Anim. Physiol. Anim. Nutr..

[bib0026] Mao H., Chen J., Zhang J., Zhang X., Xu S., Zhang L. (2024). High-energy and high-amino acid diet enhances production performance and antioxidant capacity in yellow-feathered broilers under heat stress. Poult. Sci..

[bib0027] Miao L.P., Yuan C., Dong X.Y., Zhang X.Y., Zhou M.Y., Zou X.T. (2017). Effects of dietary L-arginine levels on small intestine protein turnover and the expression of genes related to protein synthesis and proteolysis of layers. Poult. Sci..

[bib0028] Miri B., Ghasemi H.A., Hajkhodadadi I., Khaltabadi Farahani A.H. (2022). Effects of low eggshell temperatures during incubation, in ovo feeding of L-arginine, and post-hatch dietary guanidinoacetic acid on hatching traits, performance, and physiological responses of broilers reared at low ambient temperature. Poultry Sci.

[bib0029] Murakami A.E., Silva L.M.S., Fernandes J.I.M., Silveira T.G.V., Garcez Neto A.F. (2012). The effect of arginine dietary supplementation in broiler breeder hens on offspring humoral and cell-mediated immune responses. Braz. J. Poult. Sci..

[bib0030] Ndunguru S.F., Reda G.K., Csernus B., Knop R., Gulyás G., Szabó C., Czeglédi L., Lendvai Á.Z. (2024). Embryonic methionine triggers post-natal developmental programming in Japanese quail. J. Comp. Physiol. B.

[bib0032] Oliveira C.H., Dias K.M.M., Bernardes R.D., Diana T.F., Rodrigueiro R.J.B., Calderano A.A., Albino L.F.T. (2022). The effects of arginine supplementation through different ratios of arginine:lysine on performance, skin quality and creatine levels of broiler chickens fed diets reduced in protein content. Poult. Sci..

[bib0033] Pirsaraei Z.A., Rahimi A.I., Deldar H.I., Sayyadi A.J., Ebrahimi M.I., Shahneh A.Z., Shivazad M., Tebianian M. (2018). Effect of feeding arginine on the growth performance, carcass traits, relative expression of lipogenic genes, and blood parameters of Arian broilers. Braz. J. Poult. Sci..

[bib0034] Reda G.K., Ndunguru S.F., Csernus B., Knop R., Lugata J.K., Szabó C., Czeglédi L., Lendvai Á.Z. (2024). Dietary restriction reveals sex-specific expression of the mTOR pathway genes in Japanese quails. Sci. Rep..

[bib0035] Robbins K.R., Saxton A.M., Southern L.L. (2006). Estimation of nutrient requirements using broken-line regression analysis1. J. Anim. Sci..

[bib0036] Ruan D., Fouad A.M., Fan Q.L., Huo X.H., Kuang Z.X., Wang H., Guo C.Y., Deng Y.F., Zhang C., Zhang J.H., Jiang S.Q. (2020). Dietary L-arginine supplementation enhances growth performance, intestinal antioxidative capacity, immunity and modulates gut microbiota in yellow-feathered chickens. Poult. Sci..

[bib0038] Uyanga V.A., Sun L., Liu Y., Zhang M., Zhao J., Wang X., Jiao H., Onagbesan O.M., Lin H. (2023). Effects of arginine replacement with L-citrulline on the arginine/nitric oxide metabolism in chickens: an animal model without urea cycle. J. Anim. Sci. Biotechnol..

[bib0039] Uyanga V.A., Wang M., Tong T., Zhao J., Wang X., Jiao H., Onagbesan O.M., Lin H. (2021). L-citrulline influences the body temperature, heat shock response, and nitric oxide regeneration of broilers under thermoneutral and heat stress conditions. Front. Physiol..

[bib0040] Wang R., Li K., Sun L., Jiao H., Zhou Y., Li H., Wang X., Zhao J., Lin H. (2022). L-arginine/nitric oxide regulates skeletal muscle development via muscle fibre-specific nitric oxide/mTOR pathway in chickens. Anim. Nutr..

[bib0041] Westreicher-Kristen E., Davin R., Agostini P., Saremi B. (2025). Effect of different arginine-to-lysine ratios and guanidinoacetic acid supplementation on growth performance, carcass characteristics, and breast myopathies in broiler chickens. Livest. Sci..

[bib0043] Xia Z., Huang L., Yin P., Liu F., Liu Y., Zhang Z., Lin J., Zou W., Li C. (2019). L-arginine alleviates heat stress-induced intestinal epithelial barrier damage by promoting expression of tight junction proteins via the AMPK pathway. Mol. Biol. Rep..

[bib0044] Xu Y.Q., Guo Y.W., Shi B.L., Yan S.M., Guo X.Y. (2018). Dietary arginine supplementation enhances the growth performance and immune status of broiler chickens. Livest. Sci..

[bib0045] Yang L., He T., Xu Y., Zang H., Wang J., Lin Z., Jin S., Geng Z. (2019). Association analysis between feed efficiency and expression of key genes of the avTOR signaling pathway in meat-type ducks. Mol. Biol. Rep..

[bib0046] Yu J., Yang H., Wang Z., Dai H., Xu L., Ling C. (2018). Effects of arginine on the growth performance, hormones, digestive organ development and intestinal morphology in the early growth stage of layer chickens. Ital. J. Anim. Sci..

[bib0047] Yuan C., Ding Y., He Q., Azzam M.M.M., Lu J.J., Zou X.T. (2015). L-arginine upregulates the gene expression of target of rapamycin signaling pathway and stimulates protein synthesis in chicken intestinal epithelial cells. Poult. Sci..

[bib0048] Zampiga M., Laghi L., Petracci M., Zhu C., Meluzzi A., Dridi S., Sirri F. (2018). Effect of dietary arginine to lysine ratios on productive performance, meat quality, plasma and muscle metabolomics profile in fast-growing broiler chickens. J. Anim. Sci. Biotechnol..

